# Effectiveness of conversational script optimization by intelligent consultation robots on daily work efficiency in vaccination clinics

**DOI:** 10.3389/fpubh.2025.1535270

**Published:** 2025-06-09

**Authors:** Bei Zhou, Yan Xu, Jirong Shi, Yi Xiang, Daifei Chen, Qingkui Yao, Yinjun Hu

**Affiliations:** ^1^Minglou Street Community Health Service Center, Yinzhou, Ningbo, China; ^2^Shensu Science and Technology (Suzhou) Co., Ltd., Suzhou, Jiangsu, China; ^3^Ningbo Yinzhou District Center for Disease Control and Prevention, Ningbo, China

**Keywords:** intelligent consultation robot, vaccination services, conversational script impact, dialogue guidance strategy, work efficiency, patient satisfaction

## Abstract

**Introduction:**

Vaccines are essential for reducing infectious disease incidence, but challenges like public awareness and healthcare workloads persist. This study aimed to evaluate the impact of optimizing conversational scripts of an intelligent consultation robot on enhancing operational efficiency in vaccination clinics.

**Methods:**

A pilot project was conducted at the vaccination clinic of Minglou Street Community Health Service Center in Ningbo’s Yinzhou District. The robot system, developed by Shensu Science and Technology, was implemented from January to May 2024 with four experimental phases. The study used a pre-post comparison framework to assess changes in labor costs, work efficiency, and user satisfaction.

**Results:**

As the scripts evolved, there was a notable increase in automated response rates and a decrease in human support transfers. User satisfaction improved, particularly in the final phase. The robot became more effective at managing user inquiries, reducing reliance on manual services.

**Discussion:**

Optimizing the robot’s conversational scripts significantly improved daily operational efficiency in the vaccination clinic. By automating routine consultation tasks, the robot reduced healthcare professionals’ workloads. Future research could explore further refinements to dialogue strategies and expand the robot’s applications in healthcare settings.

## 1 Introduction

Vaccines are an effective intervention for reducing the incidence and mortality rates of certain infectious diseases (1–3). However, the implementation of vaccination programs is not without its challenges. In the current medical environment, healthcare professionals face numerous obstacles, particularly in the context of preventive vaccination services. One of the primary issues is the inadequate public understanding of the importance and benefits of vaccination (4, 5). This lack of awareness can lead to hesitancy and resistance, which in turn affects the overall success of vaccination campaigns (6, 7). In China, due to limited public awareness and the absence of a systematic adult vaccination recommendation program, adult vaccination rates remain low (8, 9). Du et al. (10) emphasized the need to improve public access to specialized vaccine-related information sources. Vaccination clinics, as primary venues for preventive services, handle daily tasks such as appointment scheduling, vaccine reminders, information inquiries, and post-vaccination guidance, which are both complex and repetitive (11). The heavy workload of healthcare workers often leaves them with limited time and capacity to effectively convey the critical information and significance of vaccinations to patients (12). In addition, inadequate medical staff expertise can also lead to the transmission of misinformation, which can lead to unnecessary delays or missed vaccinations (13).

Effective vaccination services not only ensure that a greater number of individuals receive the necessary vaccinations but also enhance patient experience, thereby fostering increased trust in healthcare systems. In this context, the rapid development of artificial intelligence (AI), particularly in speech recognition and voice interactive technologies, has marked significant advancements in the healthcare sector (14, 15). These intelligent human-computer interaction technologies have the potential to automate routine tasks, such as providing health consultations (16–18), auxiliary diagnosis (19, 20), and efficient information collection (21). This progress is not just a global trend but is also evident in China, where the integration of such technologies is transforming the delivery of healthcare services (22, 23). Studies have also attempted to improve vaccination clinic services, such as addressing vaccine hesitancy (24) and enhancing reminders (25, 26), through intelligent consultation robots. By doing so, they can free up medical staff to concentrate on more complex tasks and direct patient care. However, existing studies predominantly emphasize robotic technology development and preliminary effectiveness evaluations. There is a lack of in-depth analysis and research on the specific impact of intelligent consultation robot discourse and its actual application effects in vaccination clinics.

This paper aims to fill this gap by conducting a pilot project of the intelligent consultation robot system at the vaccination clinic of Minglou Street Community Health Service Center in Yinzhou District, Ningbo City (Minglou Clinic). This study documents the iterative process of the robot’s dialogue design and detailed analysis of user interaction logs. By analyzing the call records from January 2024 to May 2024, this study evaluates how the robot’s conversational script impacts medical staff workload, workflow efficiency, and patient satisfaction. The results of the study will provide empirical support for the application of intelligent consultation robots in vaccination clinics and provide references for further improving the quality and efficiency of preventive vaccination services.

## 2 Materials and methods

### 2.1 Intervention and study design

The intelligent consultation robot system, developed by Shensu Science and Technology (Suzhou) Co., Ltd., was designed for vaccination clinics to streamline consultation workflows, particularly by managing inbound calls from users inquiring about vaccination services. The system employs a scripted dialogue framework to interact with users, prompting them to categorize their inquiries (e.g., vaccine types, clinic schedules) and delivering tailored responses based on their input. The system comprises four core components: (1) a speech recognition module that transcribes voice inputs into text; (2) a natural language processing (NLP) engine that interprets user intent and generates responses; (3) a curated knowledge base containing vaccination schedules, clinic hours, and vaccine availability data; and (4) a dialogue management system that orchestrates interactions between modules to guide users through inquiry resolution.

**Figure 1:**
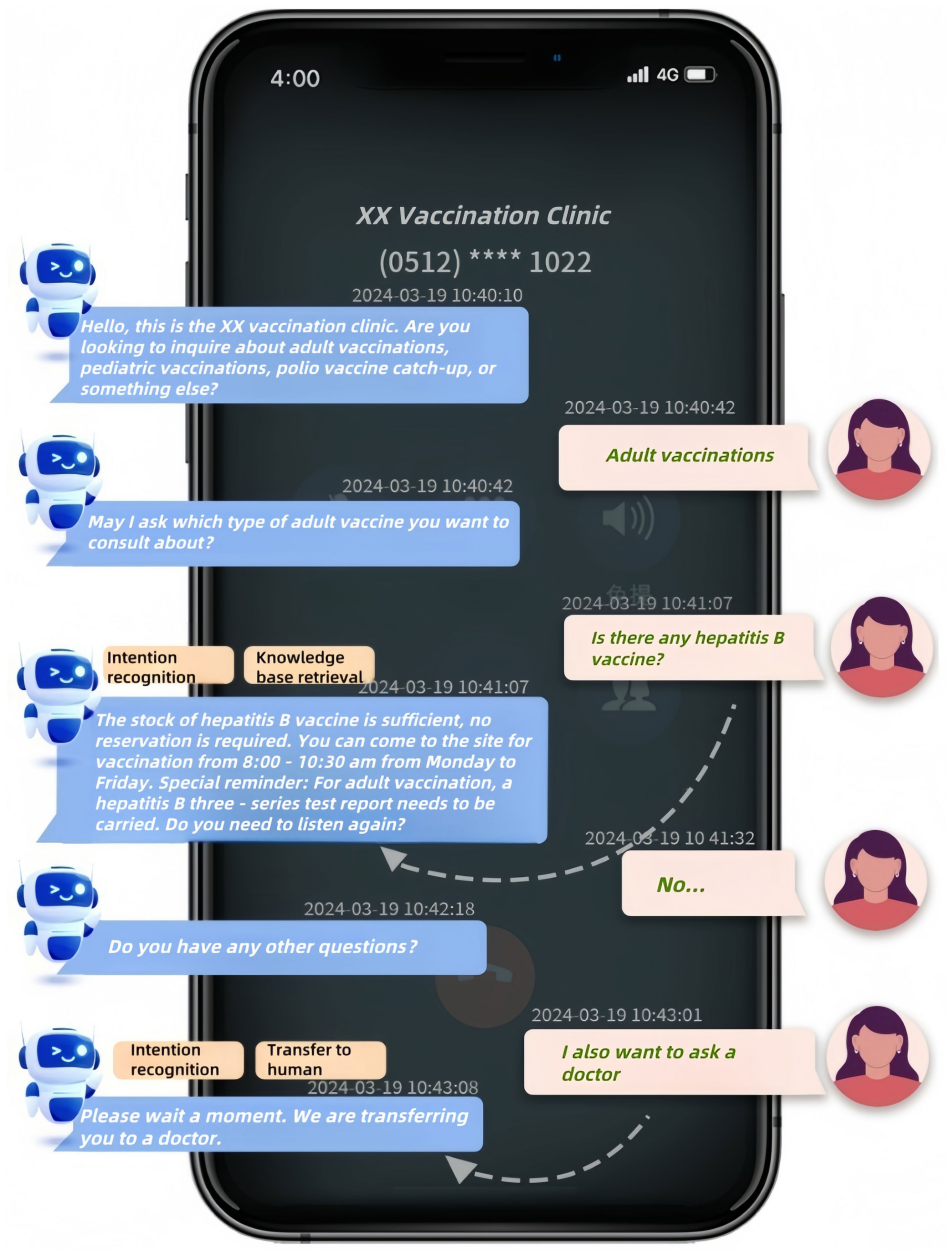
Example Interaction with the intelligent consultation robot system in a vaccination clinic. Dialogue translated from Chinese for clarity.

Figure 1 demonstrates a sample dialogue flow between a user and the system (translated from Chinese to English for clarity), highlighting key stages: intent recognition, knowledge base retrieval, and transfer to human. The interaction begins with the robot greeting the user and requesting them to specify their inquiry category (e.g., “adult vaccinations” or “pediatric vaccinations”). If the user’s input is ambiguous, the system employs a clarification loop to narrow the query scope before retrieving relevant information from its knowledge base. The dialogue persists until the user’s query is fully addressed or escalation to human agents occurs after predefined thresholds (e.g., three consecutive unrecognized inputs) are met.

In this study, the robot system was customized exclusively for Minglou Clinic to optimize vaccination consultation workflows and address previously unmet challenges in preventive care services. The conversational scripts were synthesized by analyzing prevalent inquiry patterns at Minglou Clinic, integrating NLP-driven intent classification frameworks and empirical insights from prior vaccination consultation projects. Historical consultation data from the clinic and feedback from medical staff were collected and analyzed to categorize prevalent inquiries and pinpoint essential information requirements. Iterative script revisions were conducted through interdisciplinary team discussions, with versions optimized based on user interaction analytics.

A modified pre-post comparison framework was adopted to assess iterative dialogue guidance strategies in vaccination clinic operations. All experimental phases (Experimental 1–4) were conducted between January and May 2024. Experimental 1 established a baseline post-deployment script, while Experimental 2–4 introduced optimized versions. Each phase targeted distinct operational metrics (efficiency, costs, satisfaction), with outcomes compared across groups to quantify incremental gains. Key features of the dialogue guidance strategies are summarized in Table 1 (complete Chinese scripts in Supplementary Table S1):

1. Experimental 1 (Baseline, Jan 5–18): Open-ended single-round questioning with example prompts
  - In Experimental 1, the robot employs an open-ended approach to the initial round of guidance, allowing users to freely express their inquiries without being confined to specific categories. This method provides examples such as “outpatient clinic hours,” “HPV 9-valent vaccine appointments” and “printing of return-to-school certificates” to guide users in formulating their questions, yet it remains flexible to accommodate a wide range of user needs that may not be listed. However, this strategy could generate a wide array of diverse inputs, complicating the robot’s task of effectively categorizing and responding to user needs.
2. Experimental 2 (Feb 15-Mar 21): Two-round structured categorization (adult/child vaccines, clinic info)
  - Experimental 2 introduces a more structured approach by categorizing consultation questions into three main categories during the first round of guidance: adult vaccine issues, pediatric vaccine issues, and clinic information inquiries. This method requires users to select from predefined categories, prompting them to specify their inquiry category, such as “pediatric vaccines.” For inquiries that do not fit into these main categories, users are prompted to directly state keywords, like “printing of return-to-school certificates” or “HPV 9-valent vaccine appointments.”
3. Experimental 3 (Mar 26-Apr 1): Refined categorization emphasizing polio vaccine boosters
  - Experimental 3 further refines the categorization by replacing “clinic information inquiries” with “polio vaccine boosters” during the first round of guidance, placing a greater emphasis on specific vaccination-related inquiries. In this version of the conversational script, users are prompted to select between “adult vaccination issues,” “pediatric vaccination issues,” “polio vaccine boosters,” or “other.” The guiding phrases have become more concise compared to the previous version. General clinic information inquiries are now included under the “other” category and are addressed in the second round of guidance.
4. Experimental 4 (Apr 2-May 6): Reactive human service transfer protocol
  - In Experimental 4, the robot no longer proactively suggests transferring to human service during the second round of guidance; instead, it offers the option to transfer to human service immediately in two scenarios: when it cannot understand the user’s question or when the user explicitly requests a transfer to human service. This change represents a shift toward a more reactive approach, prioritizing user experience by providing immediate assistance when the robot’s understanding is challenged, rather than following a predetermined script.

**Table 1:** Robot dialogue script iteration overview.

Experimental	Setting time	Robot’s first round of questions	User’s intended responses	Robot’s second round of questions
1	January 5, 2024 (for 14 days, 2 weeks)	Hello, this is the vaccination clinic at Minglou Street Community Health Service Center. How can I help you today? Are you looking for information on our clinic’s opening hours, booking for the 9-valent HPV vaccine, or perhaps need assistance with printing a return-to-school certificate?	Clinic’s opening hours	/
2	February 15, 2024 (for 35 days, 5 weeks)	Hello, this is the vaccination clinic at Minglou Street Community Health Service Center. Are you calling to inquire about adult vaccines, pediatric vaccines, or clinic information? If your inquiry falls into one of these three categories, please specify the category by saying, for example, ‘pediatric vaccines’. If your question does not belong to any of these categories, you can also directly state the key term of your inquiry, such as ‘printing of return-to-school certificates’ or ‘booking for HPV 9-valent vaccine’ and so on.	Clinic information	May I ask which aspect of our clinic you would like to inquire about? You can ask me about ‘clinic hours’, ‘clinic address’, ‘parking at the clinic’, or ‘vaccination on weekends’. You can also inquire about keywords such as ‘medical insurance payment’, ‘medical insurance across cities’, or ‘relocation from other places’. If you need to consult about other departments, please say ‘duty room phone’ or ‘pediatric health department phone’. Is there anything else you would like to inquire about? If you need to speak with a human assistant, please say ‘transfer to a human’.
3	March 26, 2024 (for 7 days, 1 week)	Hello, this is the vaccination clinic at Minglou Street Community Health Service Center. Are you looking to inquire about adult vaccinations, pediatric vaccinations, polio vaccine catch-up, or something else?	Something else	Alright, for other inquiries, here are some options you can choose from by stating your question: ‘clinic information’, ‘medical insurance payment’, ‘pediatric health department phone’, ‘general duty room phone’, ‘postponing vaccination’, and so on. Is there anything else you would like to consult about? If you need to speak with a human operator, please say ‘transfer to human’.
4	April 2, 2024 (for 35 days, 5 weeks)	Hello, this is the vaccination clinic at Minglou Street Community Health Service Center. Are you looking to inquire about adult vaccinations, pediatric vaccinations, polio vaccine catch-up, or something else?	Something else	Alright, for other inquiries, here are some options you can choose from by stating your question: ‘clinic information’, ‘medical insurance payment’, ‘pediatric health department phone’, ‘general duty room phone’, ‘postponing vaccination’, and so on.

**Figure 2:**
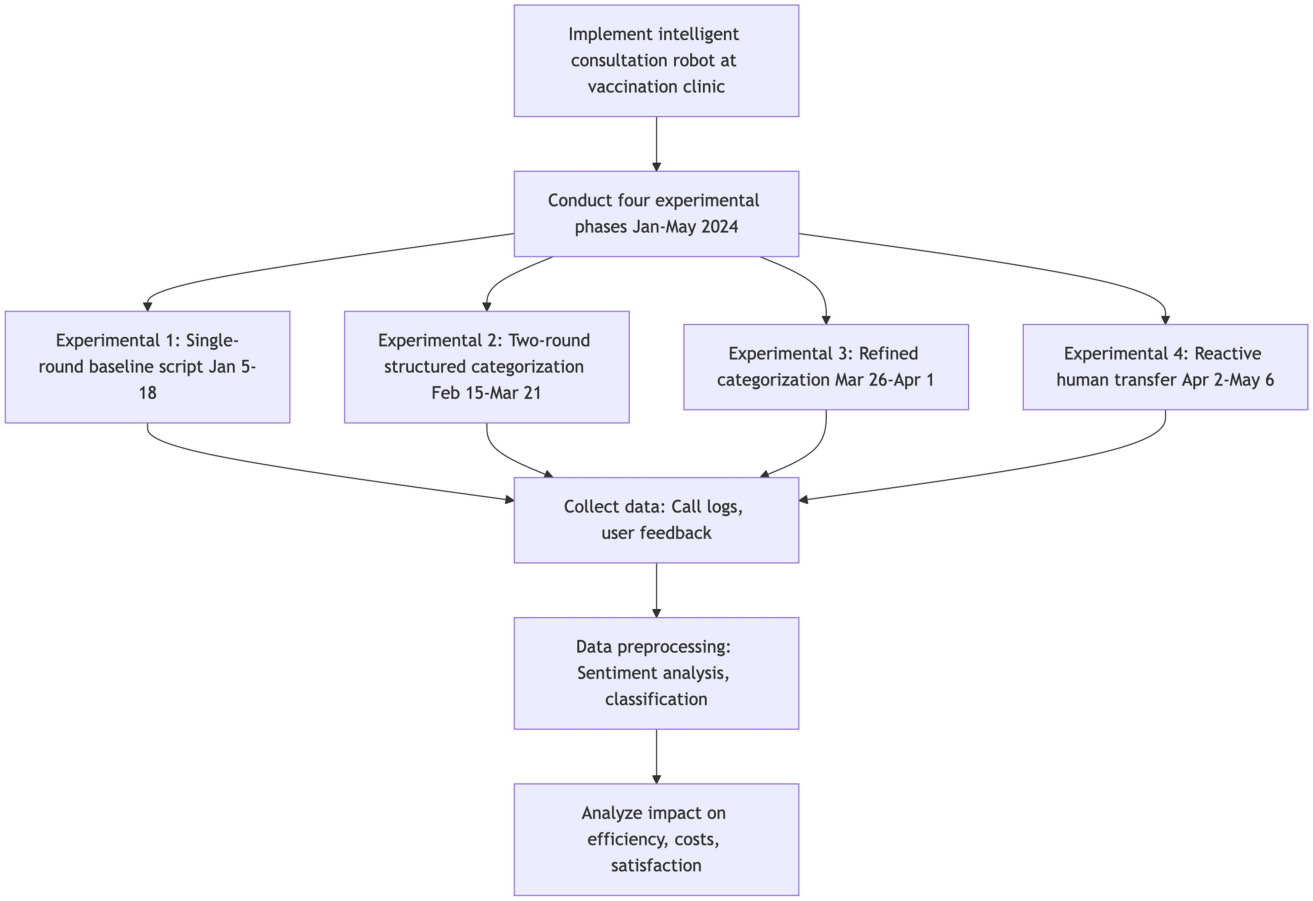
Research flowchart: assessing the impact of conversational script optimization on operational efficiency in a vaccination clinic.

Each script iteration underwent a 7–35 day field trial (total *n* = 1,076 calls), with system logs capturing full interaction transcripts, resolution pathways, and user feedback. Figure 2 presents the entire research process, from the implementation of the intelligent consultation robot system at the vaccination clinic, through the four experimental phases, to the data collection, processing, and final analysis.

### 2.2 Data collection and processing

Call records were collected between January 5 and May 6, 2024, excluding two experimental phases (January 19 and March 22) due to insufficient valid user interactions (<50 calls). Only real user inquiries (excluding system-generated simulations) with complete metadata (timestamp, query type, and resolution status) were included. The dataset includes four experimental phases (Experimental 1–4) with sample sizes range from 96 to 427 annotated interactions, as detailed in Table 2.

**Table 2:** Summarized dataset.

Experimental	Duration	Calls	Mean calls/Day (standard deviation)
1	Jan 5–18	204	14.6 (4.7)
2	Feb 15–Mar 21	427	13.8 (8.0)
3	Mar 26–Apr 1	96	13.7 (7.4)
4	Apr 2–May 6	349	10.0 (7.0)

Data preprocessing included sentiment labeling according to the following criteria:

- Whether the call was hung up immediately after being answered
- Whether the caller was satisfied with the assistance
- Whether the call was directly transferred to human service
- Whether the call was fully handled by the robot without transfer
- The number of routine questions contained in the call
- The number of routine questions resolved by the robot during the call.

Two complementary methods assessed caller satisfaction: (1) direct validation through a post-conversation survey where users rated their experience via button presses or voice commands, and (2) indirect inference via real-time conversational analysis detecting positive expressions (e.g., “Thank you,” “Very satisfied”) and negative cues (e.g., “It’s a mess,” “Do not talk anymore”).

Routine questions referred to inquiries about vaccination protocols and clinic information preloaded into the robot’s knowledge base.

### 2.3 Outcome measures

This study examined the impact of conversational script iterations across three key dimensions: labor costs, work efficiency, and user satisfaction.

#### 2.3.1 Labor costs

To assess the labor cost savings from the intelligent consultation robot, two primary indicators are utilized: the fully automated response rate and the direct human support transfer rate:

The fully automated response rate $=\frac{n_{\text{auto}}\;}{\text{Total calls}}$

The direct human support transfer rate $=\frac{n_{\text{transfer}}}{\text{Total calls}}$

where $n_{\text{auto}}$ represents the number of calls fully handled by the robot, and $n_{\text{transfer}}$ represents the number of calls in which users choose to directly transfer to human service without following the robot’s guidance process.

#### 2.3.2 Work efficiency

The study indirectly assessed the robot’s impact on the work efficiency of medical staff by monitoring two metrics: the average number of routine questions per call and the average number of routine questions resolved per call:

The average number of routine questions per call $=\frac{n_{\text{routine}}\;}{\text{Total calls}-n_{\text{hangup}}}$

The average number of routine questions resolved per call $=\frac{n_{\text{resolved}}\;}{\text{Total calls}-n_{\text{hangup}}}$

where $n_{\text{routine}}\;$ represents the number of routine questions, $n_{\text{resolved}}$ represents the number of routine questions being resolved by robot in calls, and $n_{\text{hangup}}$ represents the number of calls that was hung up immediately.

#### 2.3.3 User satisfaction

For user satisfaction, the number of satisfied calls and the number of unsatisfied calls were calculated for each experimental phase. Additionally, the number of calls that had no satisfaction assessment recorded was also calculated.

### 2.4 Statistical methods

The data analysis was conducted using Python 3.11.5 and R 4.3.2, employing a combination of statistical methods to evaluate the performance metrics and user satisfaction. For trend analysis, least squares smoothing was applied to the weekly metrics, which facilitated the identification and visualization of underlying patterns and trends in the data over time. To compare the performance metrics across different experimental phases, independent t-tests were conducted. For the satisfaction analysis, Fisher’s Exact Test was applied to the categorical feedback data presented in a contingency table, allowing for a robust assessment of differences in user satisfaction between experimental phases.

## 3 Results

### 3.1 Labor costs

**Figure 3:**
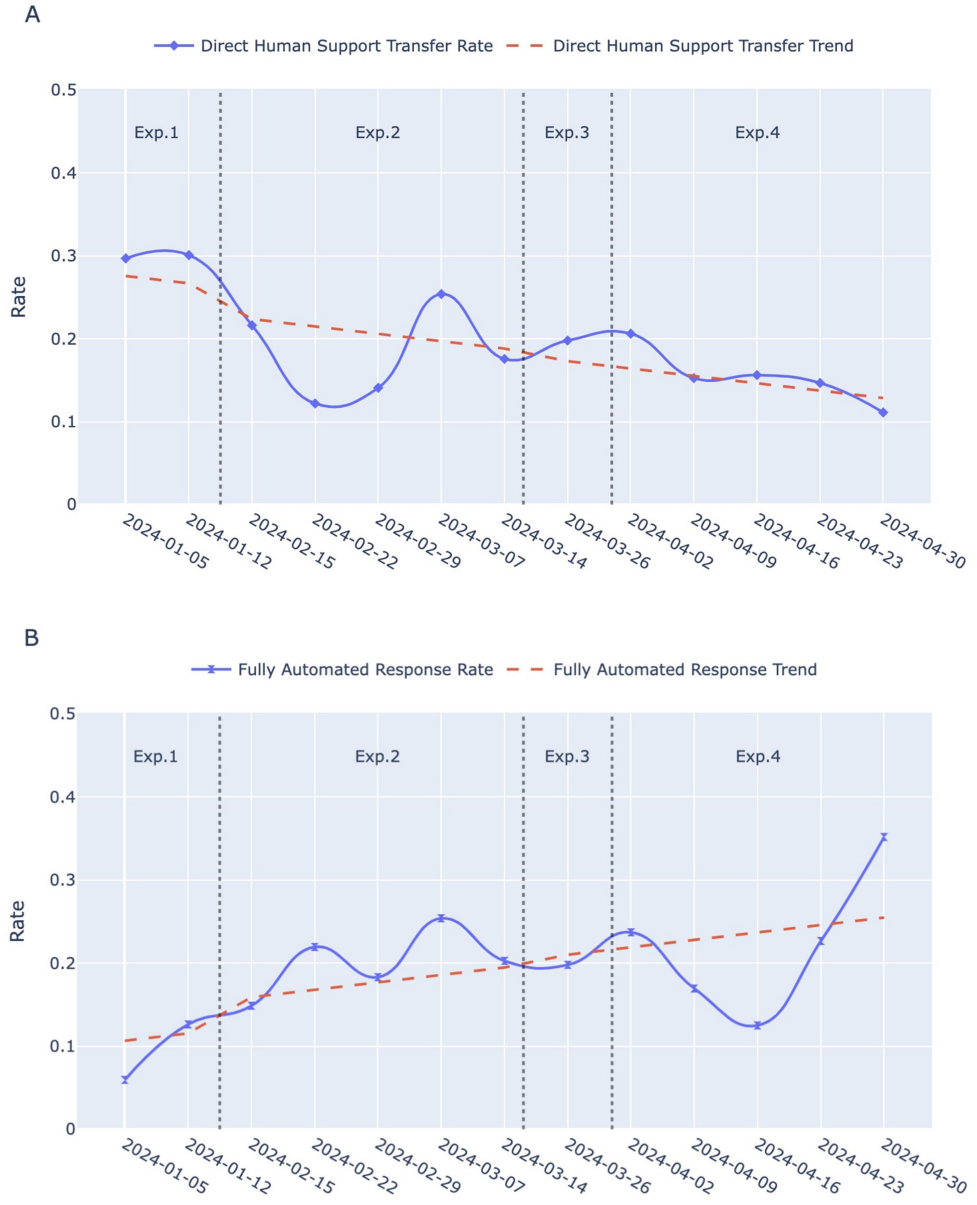
Temporal trends in two indicators across Exp. 1–4: Experimental 1–4. **(A)** Direct human support transfer rates over weeks for the four experimental phases. The blue solid line with diamond markers represents the weekly rates, while the red dashed line indicates the smoothed trend. **(B)** Fully automated response rate over weeks for the four experimental phases. The blue solid line with hourglass markers indicates the weekly rates, and the red dashed line represents the smoothed trend.

**Figure 4:**
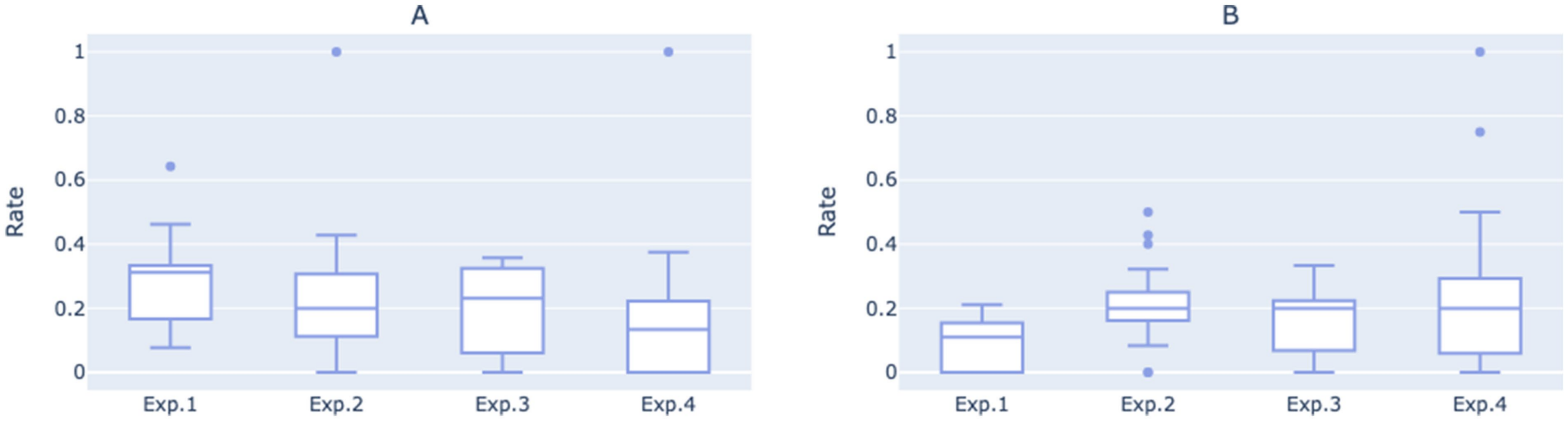
**(A)** Boxplot of direct human support transfer rates for each of the four experimental phases based on daily statistics. **(B)** Boxplot of fully automated response rate for each of the four experimental phases based on daily statistics.

Figure 3 displays the fully automated response rate and direct human support transfer rates for each of the four experimental phases on a weekly basis, whereas Figure 4 presents boxplots of these rates for the same experimental phases, based on daily statistics. The least squares method was utilized to compute the trend lines of the data, shown as the red dashed lines in Figure 3. These lines indicate an overall declining trend in the direct human support transfer rate and an overall increasing trend in the fully automated response rate as the conversational script iterates.

In the early period of Experimental 1 (from Jan 5 to Jan 11), the direct human support transfer rate stood at 30, and 12% of the calls were hung up immediately after being connected. Consequently, the open-ended, single-round guidance script engaged only about 58% of users in interactions with the robot effectively. The higher variability in user questions also indicates that the robot struggled to address them effectively.

Transitioning into Experimental 2, the direct human support transfer rate decreased, while fluctuations in the fully automated response rate showed an upward trend. An independent t-test revealed a statistically significant improvement in the fully automated response rate compared to Experimental 1 (*p* = 0.0030), suggesting that the rapid categorization of structured Q&A models is better suited for the workflows in vaccination clinic.

Experimental 3 introduced a more concise guidance script, leading to a further increase in the fully automated response rates, while the median daily direct human support transfer rate showed a slight upward trend.

In Experimental 4, after ceasing to explicitly prompt for human service, the direct human support transfer rate gradually dropped to 11% during the last week of the study (from April 30 to May 6). However, the fully automated response rate experienced a sudden decline. Nevertheless, it did not continue to decline; instead, it began to rise during the week commencing April 23 and eventually reached 35%. This unexpected reversal might be due to users adapting to the implicit handoff mechanism, as the removal of explicit prompts encouraged initial exploration of automated features before stabilizing at a higher efficiency level. Notably, the improvements in both the direct human support transfer rate (*p* = 0.0070) and the fully automated response rate (*p* = 0.0047) from Experimental 1 to Experimental 4 were statistically significant.

### 3.2 Work efficiency

When the intelligent consultation robot can effectively handle simple inquiries, it allows medical staff to focus on resolving complex and unconventional issues. As a result, medical personnel will no longer need to spend time explaining common questions, which is expected to reduce the duration of each telephone consultation. This ensures that the calls medical staff receive truly require their professional knowledge and skills, thereby reducing the time spent on phone calls and improving work efficiency while optimizing resource allocation. Furthermore, if the robot’s dialogue guidance is well-designed and effective, it will help users clarify their thoughts and define their issues before being transferred to human service. When users can express their needs and problems more accurately, it will also enhance the efficiency of communication after the transfer.

**Figure 5:**
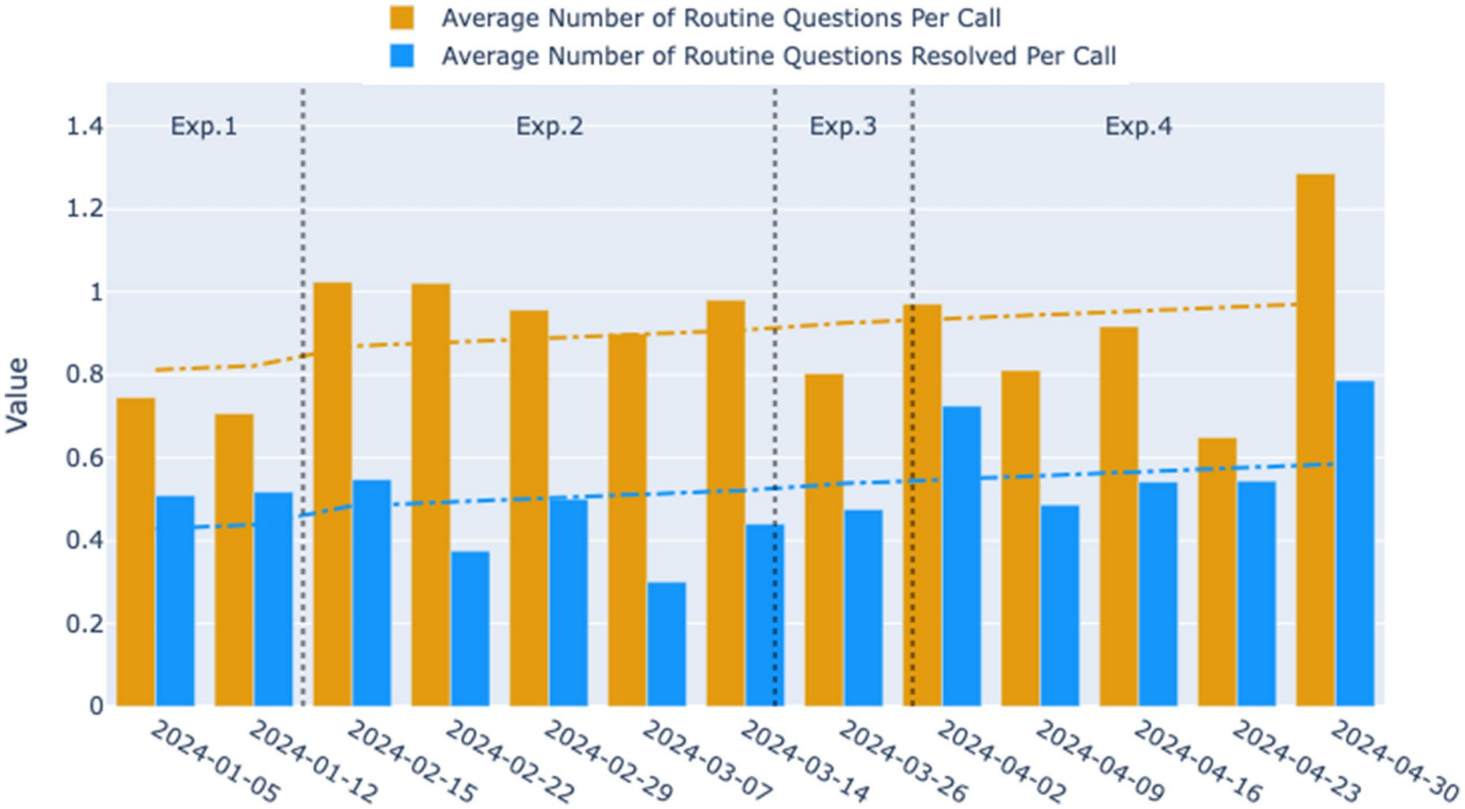
Weekly trends in the average number of routine questions per call and the average number of routine questions resolved per call for the four experimental phases. The yellow bars represent the average number of routine questions per call, and the blue bars indicate the average number of routine questions resolved per call across the observed weeks. The dashed lines represent the smoothed trend lines, illustrating the overall direction of the data over time.

As shown in Figure 5, the average number of routine questions per call was approximately 0.75 during the time of Experimental 1, indicating that the relatively open-ended mode was not adept at guiding users to pose routine questions or helping them clarify their inquiry objectives. Users’ inability to extract effective information from the initial prompts may have contributed to a higher direct transfer to human rate.

**Figure 6:**
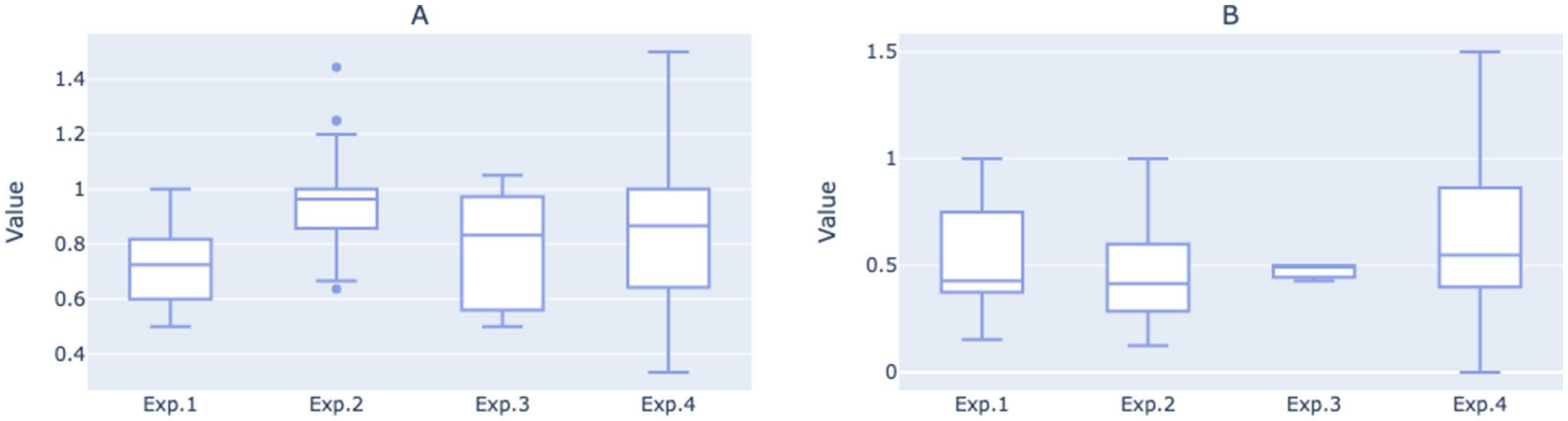
**(A)** Boxplot of average number of routine questions per call for each of the four experimental phases based on daily statistics. **(B)** Boxplot of average number of routine questions resolved per call for each of the four experimental phases on daily statistics.

When transitioning to Experimental 2, the average number of routine questions per call initially rose to 1.02, followed by a gradual decline to 0.9 over time. However, the average remained significantly higher than that in Experimental 1 (*p* = 0.0018), suggesting that the two-round dialogue guidance system in Experimental 2 more effectively facilitated users in clarifying and articulating their questions. Despite the increase, the number of routine questions resolved per call did not see a significant improvement, instead exhibiting a fluctuating decline. This trend is evident upon examining the median values of the daily statistics for the two experimental phases (Figure 6). It suggests that during the second phase, while more questions were raised by users, the dialogue may not have been standardized or clear enough to ensure that all questions were adequately addressed during the phone interactions.

After entering the third and fourth iterations, the average number of routine questions resolved per call showed an upward trend, eventually reaching 0.79. The average number of routine questions posed per call in Experimental 4 was comparable to Experimental 2, but with greater variance, peaking at 1.29. This indicates that under the structured two-round guidance system, as the dialogue script gradually adopted more concise language, the robot became more effective at resolving users’ issues during phone interactions, thereby enhancing the overall work efficiency of medical staff.

Moreover, when observing the overall growth trends for the two indicators, it was also noted that the gap between them has narrowed, especially when comparing Experimental 4 with Experimental 2, suggesting that a higher proportion of user questions were resolved during each call.

### 3.3 Customer satisfaction

Satisfaction feedback summaries for each experimental are displayed in Table 3. Due to the inability of call records to capture user emotions, the amount of clear positive or negative satisfaction evaluation data obtained solely from text is limited. Despite this, it was observed that Experimental 4 did not receive any negative satisfaction evaluations, in stark contrast to the 4 negative evaluations received by Experimental 1.

**Table 3:** Satisfaction feedback summary.

Satisfaction level	Experimental 1	Experimental 2	Experimental 3	Experimental 4
Satisfied	1	2	1	7
Dissatisfied	4	3	1	0
No Response	211	499	98	338

To further explore the differences in satisfaction between the experimental phases, Fisher’s Exact Test was employed for statistical analysis of the data from Experimental 1 and Experimental 4. The result shows a significant difference in satisfaction performance between the two experimental phases, with a *p*-value of 0.0097, indicating a significant difference in satisfaction for Experimental 4 compared to Experimental 1. This suggests that the robot’s concise and clear two-round guidance script has a positive effect on enhancing user satisfaction.

## 4 Discussion

In the scenario of incoming calls for consultation, the dialogue guidance strategy of the intelligent consultation robot is vital for enhancing the efficiency of resolving issues. If the guidance is not thorough, it may result in decreased efficiency when the robot assists users in solving problems, thereby increasing the working time required from medical staff. Conversely, accurate and clear scripts can guide users to express their issues quickly and precisely, allowing the robot to provide prompt responses. This efficient interaction not only reduces the waiting time for users but also improves the interaction experience between users and the robot, ultimately helping to increase overall customer satisfaction.

This paper elaborates on the positive impact of the robot’s precise and clear conversational scripts on the daily operations of vaccination clinics in terms of labor cost savings, work efficiency enhancement, and user satisfaction. However, this study also reveals several potential directions for future research and system improvements:

### 4.1 User satisfaction data collection

Currently, the number of satisfaction evaluations provided by users is relatively low. Future work could focus on further optimizing the satisfaction evaluation mechanism and conversational scripts to stimulate users’ interest in providing feedback (27–29), thereby collecting a greater number of direct evaluations. Additionally, applying large language models (LLMs) to perform real-time sentiment analysis (30, 31) on user voice data can help judge and record users’ emotions during conversations, serving as indirect evaluations to assist in analyzing the overall satisfaction.

### 4.2 Trade-offs in reactive human service transfer protocol

The significant improvement in fully automated response rates during Experimental 4 suggests that removing explicit “transfer to human” prompts enhanced system efficiency by encouraging users to explore automated features first. However, it risks creating “silent dropouts” of unresolved user issues. Human service was triggered only by system confusion (after three repeated queries) or explicit user requests. Users might abandon the system before reaching the threshold for human service transfer (e.g., due to impatience or low tolerance for iterative interactions). To address this issue, future designs could integrate adaptive thresholds that dynamically adjust escalation triggers based on real-time user behavior (e.g., session duration, response latency) rather than fixed repetition counts. Future work should prioritize user-centered metrics, such as issue resolution completeness and abandonment rate, to comprehensively evaluate the effectiveness of the system.

### 4.3 Post-transfer call recording and analysis

The system currently lacks the capability to record and save calls after users are transferred to human services. The absence of this feature limits the comprehensive assessment of the user service experience and the optimization of the dialogue guidance structure. With the implementation of this feature, the robot will be able to systematically collect and analyze the types, frequencies, complexities, and urgencies of the issues raised by users after being transferred to human services. This data will help identify potential bottlenecks and areas for improvement in the conversational script design. Furthermore, by analyzing the quality of the questions posed by users to medical staff, it will be possible to more accurately assess whether the conversational script iterations effectively help users clarify their inquiries and how these changes impact the daily operational efficiency of the vaccination clinic.

### 4.4 Addressing immediate call terminations

Another advantage of using an intelligent consultation robot is its ability to help the clinic filter out unwanted sales and nuisance calls, preventing the waste of medical staff’s time. During the study, about 12% of the calls were hung up immediately after being connected. Besides being potential nuisance calls, some of these calls might be users who hang up immediately upon realizing they are interacting with a robot (32, 33). On one hand, optimizing the initial conversation script and adjusting the robot’s voice intonation could improve user acceptance, reducing the hang-ups by non-nuisance users and giving the robot more opportunities to address routine inquiries, thereby reducing the burden on on-site medical staff. On the other hand, in the future, number recognition technology could be employed to effectively intercept nuisance calls, thereby reducing the likelihood of these calls being transferred to human services.

## Data Availability

The data analyzed in this study is subject to the following licenses/restrictions: the datasets analyzed during the study are not publicly available due to privacy concerns regarding the inclusion of personal identifiers such as telephone numbers, but de-identified datasets are available from the corresponding author and Minglou Street Community Health Service Center on reasonable request. Requests to access these datasets should be directed to Yinjun Hu, ssdshyj@163.com.
