# Supplementary material for: Effectiveness of conversational script optimization by intelligent consultation robots on daily work efficiency in vaccination clinics
Source: Front Public Health. 2025 Jun 9;13:1535270. doi: 10.3389/fpubh.2025.1535270 (PMC12183230; doi:10.3389/fpubh.2025.1535270)
Supplement: Supplementary file 1 [file Supplementary_file_1.DOCX]

**Table S1. Robot Dialogue Script Iteration Overview (Original Version)**

| **Experimental** | **Setting Time** | **Robot's First Round of Questions** | **User's Intended Responses** | **Robot's Second Round of Questions** |
| --- | --- | --- | --- | --- |
| 1 | January 5, 2024 (for 14 days, 2 weeks) | 您好，这里是明楼街道社区卫生服务中心接种门诊。请问您来电是想咨询哪方面的业务呢?例如，请对我说门诊开诊时间、九价疫苗预约、复课证明打印等。 | 门诊开诊时间 | / |
| 2 | February 15, 2024 (for 35 days, 5 weeks) | 您好，这里是明楼街道社区卫生服务中心接种门诊。请问您来电是想咨询成人疫苗还是儿童疫苗又或者是门诊信息？如为以上三种之一，请对我说出该咨询类别，如，“儿童疫苗”。如果您要咨询的内容不属于以上三类，您也可以直接说出要您咨询的关键词，如“复课证明打印”或者“宫颈癌九价预约”等。 | 门诊信息 | 请问您要咨询本门诊的哪方面信息。你可以问我“开诊时间“，“门诊地址”，“门诊停车”，“周末接种”，也可以咨询以下关键词，如“医保支付”、“医保跨市”或者“外地迁入”。如要咨询其他科室问题请对我说，“值班室电话”或者“儿童保健科电话”。请问您还有其他想要咨询的吗？如需转人工，请对我说转人工。 |
| 3 | March 26, 2024  (for 7 days, 1 week) | 您好，这里是明楼街道社区卫生服务中心接种门诊。请问您想要咨询成人接种问题、儿童接种问题、脊灰疫苗补种还是其他？ | 其他 | 好的，其他问题有下面这些选项，可以说出您想咨询的问题，如“门诊信息”、 “医保支付”、“儿保科电话”、“总值班室电话”、“推迟接种”等。请问您还有其他想要咨询的吗？如需转人工，请对我说转人工。 |
| 4 | April 2, 2024  (for 35 days, 5 weeks) | 您好，这里是明楼街道社区卫生服务中心接种门诊。请问您想要咨询成人接种问题、儿童接种问题、脊灰疫苗补种还是其他？ | 其他 | 好的，其他问题有下面这些选项，可以说出您想咨询的问题，如“门诊信息”、 “医保支付”、“儿保科电话”、“总值班室电话”、“推迟接种”等。 |
